# Gene cassette transcription in a large integron-associated array

**DOI:** 10.1186/1471-2156-11-82

**Published:** 2010-09-15

**Authors:** Carolyn A Michael, Maurizio Labbate

**Affiliations:** 1 Department of Biological Sciences, Macquarie University, Sydney, NSW, Australia; 2 The I3 Institute, University of Technology, Sydney, NSW, Australia

## Abstract

**Background:**

The integron/gene cassette system is a diverse and effective adaptive resource for prokaryotes. Short cassette arrays, with less than 10 cassettes adjacent to an integron, provide this resource through the expression of cassette-associated genes by an integron-borne promoter. However, the advantage provided by large arrays containing hundreds of cassettes is less obvious. In this work, using the 116-cassette array of *Vibrio *sp. DAT722 as a model, we investigated the theory that the majority of genes contained within large cassette arrays are widely expressed by intra-array promoters in addition to the integron-borne promoter.

**Results:**

We demonstrated that the majority of the cassette-associated genes in the subject array were expressed. We further showed that cassette expression was conditional and that the conditionality varied across the array. We finally showed that this expression was mediated by a diversity of cassette-borne promoters within the array capable of responding to environmental stressors.

**Conclusions:**

Widespread expression within large gene cassette arrays could provide an adaptive advantage to the host in proportion to the size of the array. Our findings explained the existence and maintenance of large cassette arrays within many prokaryotes. Further, we suggested that repeated rearrangement of cassettes containing genes and/or promoters within large arrays could result in the assembly of operon-like groups of co-expressed cassettes within an array. These findings add to our understanding of the adaptive repertoire of the integron/gene cassette system in prokaryotes and consequently, the evolutionary impact of this system.

## Background

Integrons are genetic elements capable of mobilising and rearranging genes packaged as mobile gene cassettes in a site-specific manner [1]. In concert with other mechanisms capable of mobilising DNA between cells, the integron/gene cassette system contributes to the overall process of lateral gene transfer (LGT). LGT is a major contributor to genetic diversity amongst prokaryotes [2] and hence a significant force in prokaryote evolution. The ability of the integron/gene cassette system in particular, to influence the evolution of prokaryote strains is graphically shown in the rapid dissemination of antibiotic resistance genes both geographically and amongst different prokaryotes [3]. However, cassette-associated genes are not limited to the provision of antibiotic resistance phenotypes, with a plethora of novel ORFs (open reading frames or putative genes) present in the gene cassette metagenome [4]. While the majority of the ORFs contained within gene cassettes have no analogue in sequencing databases, those few for which a function has been attributed, have been adaptive in nature [5-7].

An integron typically consists of a integrase gene (*intI*), its associated promoter P_int,_, an *attI *site which acts both as a recognition site for the integrase produced by *intI *and an insertion site for gene cassettes and a second promoter, P_c_, located within the *intI *gene but oriented towards *attC *and any adjacent gene cassette array. The DNA segments mobilised by integrons are termed gene cassettes. Gene cassettes typically consist of an ORF, closely bounded by a multifunction site termed *attC*. *AttC*, analogous to *attI *in the integron, serves as both an integrase recognition and recombination site. *AttC*, through its imperfect symmetry, also serves to orient inserted cassettes and their contained ORFs, uniformly with respect to the adjacent integron and consequently, the integron-borne promoter P_c _[8].

Site-specific recombination catalysed by the integrase, IntI, causes gene cassettes to be inserted either at *attI *or *attC *sites. Successive rounds of recombination can introduce new cassettes and so generate tandem cassette arrays adjacent to the integron. In addition, repeated recombination may also rearrange cassettes within an array. Cassette arrays can vary in length from lone integrons and the typically short (1-8 cassettes) antibiotic resistant arrays seen associated with class 1 integrons, to over 200 tandem cassettes in a single array seen in the vibrio [9,10].

Class 1 integrons were initially characterised through the antibiotic resistance phenotypes conferred through the expression of cassette-associated genes. The expression of these genes has been shown to be due to the integron associated P_c _[11]. However, it is very unlikely that P_c _could mediate the expression of all cassettes present in arrays containing significantly more than the 7-10 cassettes typically seen in class 1 integrons, due to the extreme length of the mRNA transcript required. Therefore, P_c _mediated expression alone may not account for the selective advantage provided by the presence of large gene cassette arrays containing hundreds of cassettes. It has been hypothesised that in such large arrays, only those cassettes proximal to the integron are expressed, and that the remainder of cassettes in the array are 'banked', forming an accessible population resource of mobile genes [12]. This hypothesis may be supported by the observation that under stress, the integrase gene *intI*, is up-regulated [13]. Such increased integrase activity could not only introduce or excise cassettes from the array, but also rearrange existing cassettes, so bringing previously distal cassettes closer to P_c _and hence facilitating their expression. We however, speculated that cassettes throughout large arrays were generally expressed through the presence of promoters within the array as suggested by micro-array data [14].

Resolving the question of the expression of cassette associated genes in large arrays is important in extending our understanding of the adaptive potential of gene cassettes arrays, and by extension the way in which LGT can provide varied phenotypes in prokaryote populations. In order to investigate cassette expression in large arrays, we used *Vibrio sp*. DAT722 as a model system, *Vibrio sp. *DAT722 is a weak pathogen of crustaceans and contains an integron with an attendant array of 116 gene cassettes. This array having been previously sequenced and annotated enabled a detailed examination of the differential expression of cassette-associated genes to be undertaken [15]. Therefore, in this work, the following questions were addressed:

1/ Are only cassettes proximal to the integron in *Vibrio sp*. DAT 722 expressed?

2/ If cassettes throughout the *Vibrio sp*. DAT722 array are expressed, is this due to the presence of a single long transcript or else due to multiple promoters producing many shorter transcripts?

3/ Is cassette expression, if present, conditional or constitutive and does this vary across the array?

4/ If multiple promoters are present within the *Vibrio sp*. DAT722 array, are they all the same?

## Methods

### Gene cassette expression

It has been shown that transcription of DNA into mRNA is the most limiting step in the expression of prokaryote genes, due to the general translation of mRNA by either the presence of Shine-Dalgarno type sequences, or through leaderless translation [16,17]. Accordingly, in order to address questions regarding the expression of cassette-associated genes, in this work we examined the gene cassette transcriptome of a *Vibrio *sp. DAT722.

**Figure 1:**
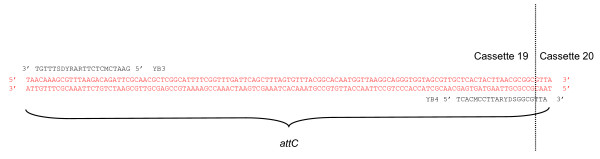
***AttC *PCR primer design**. Primers YB3 and YB4 are shown aligned with a typical *attC *sequence (the cassette 19-20 junction in this case) from the *Vibrio sp*. DAT722 array. The dotted line indicates the *attC *recombination site. YB3 is shown reversed for clarity.

**Figure 2:**
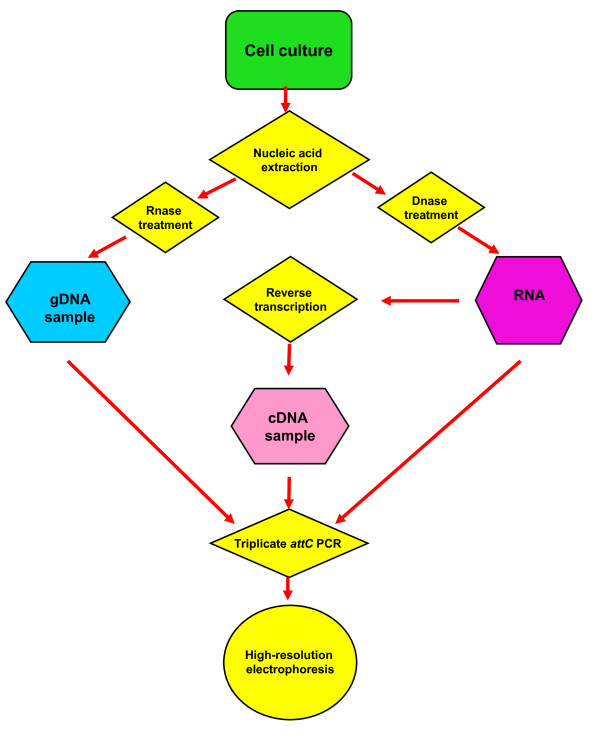
**A flow chart of the molecular methods used in this work**. Parallel extractions of gDNA and RNA from a single cell culture with subsequent reverse transcription of the RNA, allowed an analytical comparison to be made between the gDNA and cDNA gene cassette profiles by an *attC *specific PCR. The RNA sample acted as a control for gDNA contamination present in the derived cDNA sample.

**Figure 3:**
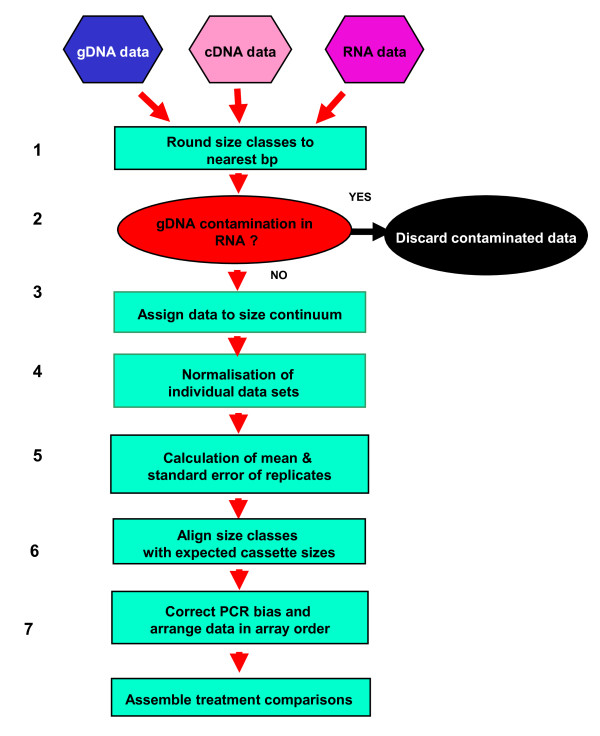
**A flow chart of the analytical process**. Individual 'size class fingerprints' were firstly, (**1**) calibrated to size (bp) and then, (**2**) Fingerprints were assessed for gDNA contamination. (**3**) Data were assigned to a size continuum and (**4**) normalised so that heights of peaks between fingerprints could be compared. (**5**) The variability in each size class was measured amongst replicate PCRs, to establish significance levels. (**6**) Amplicon peaks were correlated with expected gene cassette amplicon sizes. (**7**) PCR biases were corrected to allow comparison of peaks within a fingerprint and the peaks representing gene cassettes rearranged into their order within the gene cassette array.

The major analytical tool used in this work was a PCR that amplified a subset of all gene cassettes within a sample by using the relatively conserved areas in gene cassette-associated *attC *sites as targets (Figure 1). This '*attC *PCR' was analogous to 'gene cassette PCR', which has been used to amplify gene cassette sequences from both genomic and metagenomic samples [18-20]. Individual cassettes were identified by comparing amplicon sizes with those predicted by published sequence data. Relative quantitation of this multiple amplicon PCR was used as a rapid method of simultaneously analysing multiple cDNA samples. Flow charts for the methodology used and its analysis are seen in Figure 2 and in Figure 3.

### Culture methods and stress assays

Monoclonal stocks of *Vibrio spp*. DAT722 were grown overnight at 28°C on vibrio media plates (Per 400 ml; Casein peptone 4 g, NaCl 4 g., MgCl_2_.6H_2_O, 1.6 g. KCl, 0.4 g. pH 7.5.) and streaked to purity. The resultant single colonies were inoculated into 5 ml vibrio media liquid cultures and then incubated overnight, under an individual stressor in the dark with moderate shaking, before nucleic acid collection. In this work, different types of stressor likely to be experienced by this marine organism, which also incite different cellular responses (thermal and oxidative stress), were applied to examine the possibility of a stressor specific response within the array. Additionally, a single stressor (oxidative stress) was measured after different time-periods from application, to evaluate the possibility of temporal variations in the stressor response of the cassette array.

#### Thermal stress

Individual treatment cultures were grown at 4°C, 14°C, or 28°C, in the dark, overnight with mild shaking. Care was taken to hold each culture at its growing temperature and in the dark until immediately before the lysis step in the nucleic acid extraction.

#### Oxidative stress, 30 minute

Individual treatment cultures were grown for 12 hours at 28°C in the dark with mild shaking and then inoculated with 3% hydrogen peroxide solution (H_2_O_2_) to give final concentrations of 0, 0.9, 1.8 and 3.6 mM H_2_O_2 _and allowed to grow for a further 30 minutes at 28°C in the dark with shaking before being harvested for nucleic acid.

#### Oxidative stress, 18 hour

To 5 ml liquid cultures of *Vibrio spp*. DAT722 was added 3% hydrogen peroxide solution (H_2_O_2_) to give final concentrations of 0, 0.9, 1.8 and 3.6 mM H_2_O_2_. These cultures were then incubated for 18 hours in the dark at 28°C with mild shaking before recovery of nucleic acid.

### RNA/DNA extraction

The SV RNA extraction kit (Promega, Wisconsin USA) was used exclusively. For RNA extraction, the manufacturer's methodology was used with the following exceptions: Dual one-hour on-column DNase digests were substituted and 0.75 ml of the final culture (OD 1.1, measured at 600 nm) was used as a sample. In parallel to RNA extraction, control DNA was recovered by using the same extraction methodology with the exception that the DNase digest was replaced with an RNase digest step.

### Reverse transcription

The MMLVh-reverse transcription system (Promega, Wisconsin USA) was used according to manufacturer's specifications using YB3 primer (Table 1).

**Table 1:** *PCR primers *(Sigma Aldrich, Castle Hill, Australia)

*Name*,	*Binding site*	*Sequence*	*Usage*
YB3 6FAM	DAT722 *attC *rev	GAATCMCTCTTRARYDSTTTGT	*attC *PCR
YB4 6FAM	DAT722 *attC *fwd	TCACMCCTTARYDSGGCGTTA	*attC *PCR, Promoter isolation
MazG f	Cassette 21 ORF fwd	GTTATCTGAGTTACAAAGTC	Method validation, Promoter isolation
MazG r	Cassette 21 ORF rev	TCCTATCGGTTGTACTTAAC	Method validation, Promoter isolation
VhC 21f	Cassette 21 ORF fwd	TATGCGCCAGAGCAATCTGAACACTAT	Promoter isolation
VhC 21r	Cassette 21 ORF rev	CCGTGAATATTGGCTAGAGCACAAACA	Promoter isolation
VhC 89f	Cassette 89 ORF fwd	ATCAGAAATTGAAGACTTGC	Method validation
VhC 89r	Cassette 89 ORF rev	TGAGACATTACGCAGTTAAA	Method validation
VhC16f	Cassette 16 ORF fwd	AAAAGTCGCTCAGAAGAATA	Promoter isolation
VhC16r	Cassette 16 ORF rev	GCATTACGGATACTTGTCTT	Promoter isolation
VhC17f	Cassette 17 ORF fwd	ACTGGTCAAAATACAACCAT	Promoter isolation
VhC17r	Cassette 17 ORF rev	TACAACATCGAGCTAACAAA	Promoter isolation
VhC18f	Cassette 18 ORF fwd	GGTTTGATAGTTACGCTGAT	Promoter isolation
VhC18r	Cassette 18 ORF rev	CAACCAAATGTGATAATGAA	Promoter isolation
VhC19f	Cassette 19 ORF fwd	GAGTGCAGCAGGTTATTTAT	Promoter isolation
VhC19r	Cassette 19 ORF rev	ATACTGACCGATAACTTTGG	Promoter isolation

### *attC *PCR

Triplicate PCRs were performed on each extracted nucleic acid sample (cDNA/gDNA/RNA) as follows:

Per 50 μl reaction: 2.0 μl × sample DNA (i.e. cDNA/gDNA/RNA), 50 nM MgCl, 10 nM dNTP, 1.0 μl × 1 mg/ml Rnase, 50 pM YB3 primer, 50 pM YB4 primer, 0.3 μl. Red Hot Taq (Abgene, Surrey, UK), 5.0 μl × 10× PCR buffer (Abgene, Surrey, UK). Primers are detailed in Table 1.

#### Thermal profile

80°C hot start, 94°C for 10 minutes initial denaturation followed by between 27 to 35 cycles of 94°C 30 sec, 55°C 30 sec, 72°C 1 min 30 sec. with terminal 72°C for 10 minutes.

### Quantitative PCR (QPCR)

Quantitative PCR was performed using the Roche Light Cycler^® ^(Roche Applied Sciences, Mannheim, Germany).

Per 10 μl reaction, using 1.0 μl × sample cDNA, 40 nM MgCl, 50 pM of each primer and 1.0 μl of SYBR Green^Tm ^master mix (Roche Applied Science, Mannheim Germany) per reaction. Primers used are detailed in table 1.

#### Thermal profile

A 10-minute initial Taq activation step at 95°C followed by 35 cycles of 15 seconds at 94°C, 15 seconds at 50°C and 30 seconds at 72°C followed by a final cool down step of 10 minutes at 20°C.

### Sequencing

Sequencing of selected gene cassettes (cassettes 21 and 89) for method validation was conducted using manufacturers recommended protocols on an ABI 377 instrument.

### High-resolution polyacrylamide gel electrophoresis

The fluorescent products of the *attC *PCR, generated with 6-Carboxyfluorescein (6FAM) labelled primers, were analysed on an ABI 377 DNA sequencer by supplying 1 ul aliquots of each PCR reaction. These were run on denaturing gels prepared according to manufacturer protocols, at approximately 2500 V for 1.5 hours. The results produced single base pair resolution to 1000 base pairs (bp) and information on the relative abundance of amplicon size classes in each sample.

### Routine agarose gel electrophoresis

Samples were run on 2% TAE gels run at 100 V for 1 hour. Gels were immersed in 20 μg/ml Ethidium Bromide solution for 15-20 minutes prior to visualisation with UV light at 260 nm.

### Data analysis

Individual sets of size class data from high-resolution electrophoresis were initially examined for DNA contamination by the presence of amplicons in the RNA and H_2_0 samples. Entire data sets were discarded if contamination was evident. Resulting 'clean' cDNA data were calibrated to size (bp) and then assigned to a bp size continuum in an Excel spreadsheet. The data were normalised to allow different levels of applied stressor to be compared within an experimental series. Normalisation was accomplished using the sum of all amplicon peak heights above 90 bp in size within a PCR as an inter-PCR standard. Replicates were utilised to calculate mean and standard error to establish the variability in each size class and hence significance levels for subsequent comparisons. The published sequence of the *Vibrio sp. *DAT722 gene cassette array [14] was analysed for expected PCR fragment lengths by identifying YB3/YB4 primer binding sites with each *attC *site and then the included sequence length was measured. PCR peaks were then correlated with expected gene cassette amplicon sizes, with PCR biases such as poly-A tailing being all corrected, and the peaks representing gene cassettes rearranged in gene cassette array order. Expression values were then calculated using the known stoichiometric relationship of cassettes in the gDNA sample as a quantitative standard, and the resulting expression data presented as ratio of cDNA/gDNA peak heights for each detectable cassette size class within a treatment (Figure 3).

### Method validation

Because the methodology used in this work was novel, it was validated by comparison with established methods as follows:

• The identification of amplicon size classes with individual gene cassette species was confirmed by gel extraction and sequencing of individual amplicon size classes (cassettes 21 and 89). These sequences were then confirmed as being the target cassette.

• Replicate studies of each stage of the methodology showed that the PCR stage invoked the majority of the variability. Consequently, triplicate PCRs were included in the methodology to assess reproducibility and set significance levels for each cassette size class. Variability in the abundance of cassette specific amplicons averaged a 12% Standard Error (%SE). Conservatively, a value of 50%SE was then adopted as the maximal variability inherent in replicate amplification of a single amplicon. Accordingly, a significant change in expression at the 95% confidence level could be defined as an observed two-fold change in expression (i.e. 1.96 × 50% SE or a 100% change in expression, and this equates to a single standard deviation, and hence a minimum 95% confidence interval).

• Within-treatment quantitation was validated by relative QPCR of single RNA sample for cassettes 21 and 89. These cassettes were selected for their large difference in observed expression and also their wide spacing within the array. Results of the QPCR study were found to be comparable with that achieved in the methodology used, with a 95% confidence limit.

• Between-treatment quantitation was confirmed by relative QPCR of cassette 21 expression across all treatments of a single stressor, and the results found to be comparable with that achieved in the methodology used, within a the 95% confidence limit.

### Promoter localisation

**Figure 4:**
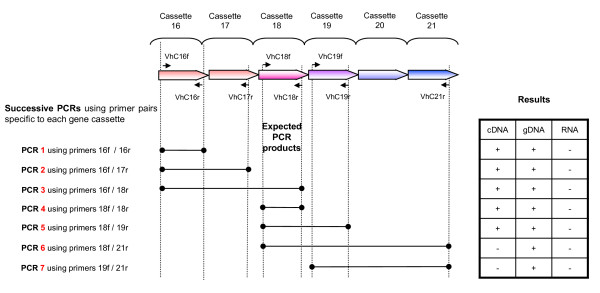
**Localising a promoter amongst cassettes 16-21 of the *Vibrio *sp. DAT722 array**. A series of PCRs using ORF-specific primers are designed to produce the products shown in the centre of the figure. Results on the right of the figure, showed that no detectable cDNA species included binding sites for both primers VhC19f and VhC21r. This indicated the presence of a promoter between these locations. Further localisation of this promoter is detailed in Figure 8.

An intra array promoter was localised through comparison PCR results on cDNA and gDNA samples, of successive nested pairs of primers initially bracketing the target area between cassettes 16 and 21 (Figure 4) and ultimately in the area encompassing cassettes 20 and 21. Promoter activity was measured by relative quantitation of QPCR amplicons both bridging and wholly downstream of the promoter.

### Promoter PCRs

Per 50 μl reaction: 2.0 μl × sample DNA, 50 nM MgCl, 10 nM dNTP, 1.0 μl × 1 mg/ml Rnase, 50 pM of each primer, 0.3 μl × Red Hot Taq (Abgene, Surrey, UK), 5.0 μl × 10× PCR buffer (Abgene, Surrey, UK). Primers are listed in Table 1.

#### Thermal profile

80°C hot start, 94°C for 10 minutes initial denaturation then between 27 to 35 cycles of 94°C 30 sec, 55°C 30 sec, 72°C 1 min 30 sec. Followed by a terminal 72°C for 10 minutes

### Promoter activity measurement by quantitative PCR (QPCR)

Quantitative PCR was performed using the Roche Light Cycler^® ^instrumentation (Roche Applied Sciences, Mannheim, Germany).

Per 10 μl reactions using 1.0 μl × sample DNA, 40 nM MgCl, 50 pM of each primer and 1.0 μl of SYBR green^Tm ^master mix (Roche Applied Science, Mannheim Germany) per reaction. PCRs using primer pairs YB4/mazGr were compared with mazGf/mazGr. Primers are detailed in table 1.

#### Thermal profile

The temperature profile included a 10-minute initial Taq activation step at 95°C per the manufacturer's recommendations, followed by 35 cycles of 15 seconds at 94°C, 15 seconds at 50°C and 30 seconds at 72°C followed by a final cool down step of 10 minutes at 20°C. PCR products were run on a 2% TAE gel to verify the presence of amplicons of expected size and the absence of non-specific amplifications.

## Results and discussion

The *Vibrio sp. *DAT722 gene cassette array contained 116 cassettes in 90 different size classes. Of these 90 size classes, 75 represented only one type of cassette, and so these size classes uniquely defined cassettes and hence their positions within the array. The remaining 15 size classes represented multiple copies of the same cassette and examples of different cassette sequences having the same length. Consequently, these 15 size classes ambiguously defined positions within the array. The methodology used in this work identified 62 of the 90 possible size classes in genomic DNA (gDNA). Of the 62 identified size classes, 50 represented unique cassettes within the array and 12 represented cassettes from amongst the 15 ambiguously located size classes. In short, 74 of the 116 cassette positions within the array were readily accessible with the techniques used in this work.

The *Vibrio sp. *DAT722 array contained 91 coding and 25 non-coding (ORF-less) cassette positions. Additionally, eight of the coding cassettes were oriented so that their genes were located on the complimentary strand. In this work, both coding cassettes and non-coding cassettes were detectably expressed. However, no expression could be specifically attributed to those cassettes with genes on the complimentary strand.

### Expression of cassette-associated genes under optimal growth conditions

**Figure 5:**
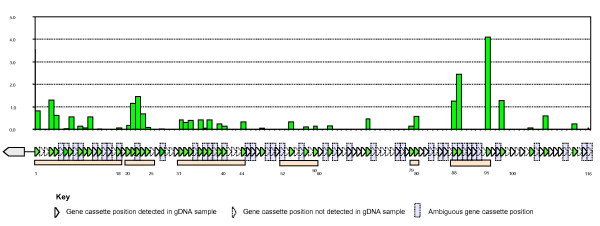
**Relative expression of gene cassettes in the *Vibrio sp*. DAT722 array**. The bar graph indicates the relative expression of each gene cassette using gDNA as an internal standard. The vertical axis indicates the level of expression while the horizontal axis indicates position within the cassette array. The schematic cassette array below the histogram indicates cassette positions within the array detected in gDNA, with solid triangles. Cassette positions not detectable with the methodology used are shown in dotted triangles. Cassettes that are ambiguously located within the array by the methodology used, due to the presence of either multiple copies of the same cassette within the array, or different cassettes of the same size, are indicated by blue rectangles. Putative expression blocs, as referred to in the text are shown by pink rectangles.

Cultures grown overnight in Vibrio media at 28^0 ^C showed that the majority of detectable cassette size classes (39 of the 62) were detectably expressed. That is, copy DNA (cDNA) species, corresponding to 39 of the 62 detectable cassettes within the array were detected in the cDNA samples. These expressed cassettes were distributed along the length of the array and were interspersed with cassettes that, while detectable in gDNA, could not be detected amongst cDNA species (Figure 5).

The technique used provided relative quantitation of expression amongst the individual gene cassettes in the DAT722 array by using the stoichiometric relationship between cassette species in gDNA as a quantitative standard. This quantitation showed at least a 100-fold difference in the intensity of expression between the most and least detectably expressed cassettes (cassettes 95 and 11 within the array respectively). Additionally, 'blocs' of adjacent expressed cassettes were expressed at similar intensities, with less than three-fold difference amongst cassettes within the bloc (for example, cassettes 31-35 in Figure 5).

The hypothesis that the integron-associated promoter, P_c _mediated expression of the entire array implies the presence of large contiguous transcripts of the array amongst cDNA species. In this work, we detected cassettes within the array whose expression was undetectable interspersed amongst detectably expressed cassettes within the array, indicating that not all of the expression seen within the array is due to P_c_. The presence of additional promoters within the array was therefore implied. Additionally, the observation that different 'blocs' of similarly expressed adjacent cassettes within the array, were expressed at levels significantly different from other 'blocs' (for example, cassettes 21-23 were significantly more expressed than the bloc containing cassettes 31-35 (Figure 5)) suggested that these additional promoters had differing abilities to catalyse transcription. The presence of detectable but unexpressed cassettes between 'blocs' indicated areas of the array where some of these 'intra-array' promoters, might be located (i.e. adjacent to blocs of cassettes; 1-18, 20-25, 31-44, 52-59 and 88-95).

### Conditional expression of gene cassette associated genes

**Figure 6:**
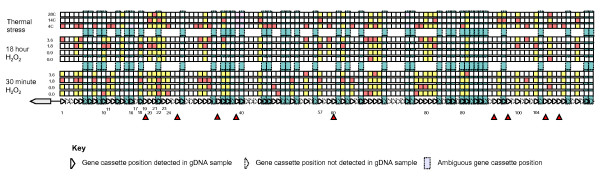
**A conditional expression map of the *Vibrio sp. *DAT722 gene cassette array under three different stressors**. The schematic cassette array below the histogram indicates cassette positions within the array detected in gDNA, with solid triangles. Cassette positions not detectable with the methodology used, are shown in dotted triangles. Cassettes that are ambiguously located within the array by the methodology used, due to the presence of either multiple copies of the same cassette within the array, or different cassettes of the same size, are indicated by blue rectangles. In the panel above the array diagram, cassettes not detectably expressed are shown in white. Detectably expressed cassettes are shown coloured in either yellow or red. Red cassette positions indicate the treatment in which maximal conditional expression was measured at the 2-fold level. Red triangles indicate the position of cassettes, which though detectable, were never measurably expressed in this work.

We then examined the question of conditional expression of cassette-associated genes in the *Vibrio sp*. DAT722 array in three experimental series. These series examined thermal stress at three levels, 4°C, 14°C and 28°C, and oxidative stress at three levels of imposed stress and a control, being 0, 0.9 mM, 1.8 mM, and 3.6 mM hydrogen peroxide, applied for two time periods, 30 minutes and 18 hours. Replicate studies showed that a two-fold change in expression both amongst cassettes within a treatment and for a single cassette between treatments in an experimental series, was significant with greater than 95% confidence. Consequently, a two-fold change in gene cassette expression was adopted, in this study, as the minimum change in expression deemed to be conditional. A summary of all gene cassette conditional expression data is shown in Figure 6.

A number of gene cassettes, detectable in gDNA were not detectably expressed under any stressor (arrowed in red in Figure 6). These cassettes were the same as those nominated as potential promoter locations in the previous section. Of the detectably expressed cassettes, all but two cassettes (23 and 70) were conditionally expressed under at least one stressor at the two-fold level of significance. The largest measured increase in expression was 11.4-fold, seen in cassette 57 under 18 hour oxidative stress, with other cassettes (eg. cassettes 10, 20 and 104 in Figure 6) showing similar levels of increase, though not always at the same level of applied stress, or even under the same stressor. Additionally, in many cases cassettes were not detectably expressed in one or more of the experimental treatments (eg. cassette 11 under both 30 minute and 18 hour oxidative stress). Consequently, the actual increase in expression of these cassettes under these stressors from undetectable levels may have been larger than that measured.

Amongst this widespread conditional expression, the following patterns were noted:

-Gene cassettes were similarly expressed within blocs. That is, within a bloc, the level of expression was largely consistent irrespective of stressor. This observation supported the suggestion that individual promoters were associated with these 'expression blocs'.

**Figure 7:**
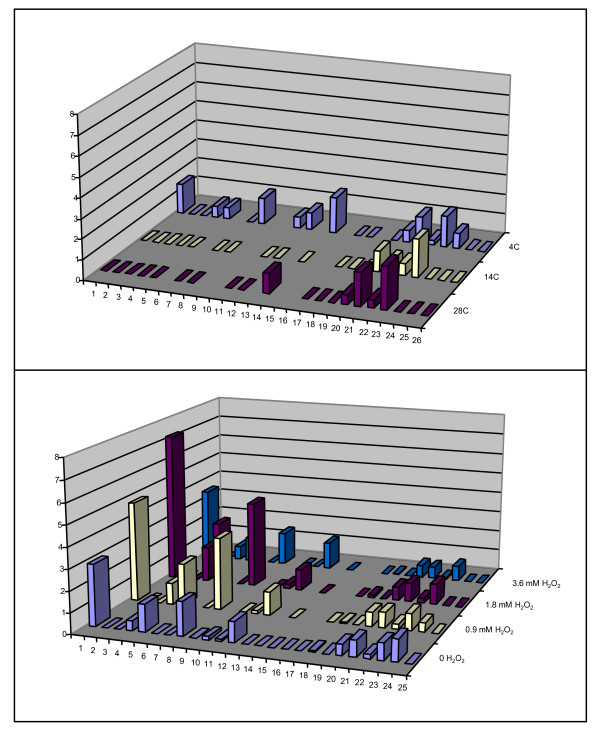
**Differential gene cassette expression under different stressors**. The lower panel shows the differential expression of the first 26 gene cassettes in the *Vibrio sp*. DAT722 cassette array under increasing 30-minute oxidative stress. The upper panel shows the same area of the cassette array under various growth temperatures. In each panel, the x-axis denotes position in the gene cassette array. The vertical axis shows the relative expression of individual gene cassettes while the depth axis shows the different expression of each gene cassette at each stressor. It was noted that cassettes 21-24 were similarly expressed under the two stressors whilst the expression of cassettes 1-15 differed markedly.

-The particular expression response to a stressor varied in both direction and extent amongst expression blocs. That is, the expression of some blocs was increased under a particular stressor whilst others blocs were not. This suggested that different types of promoter were responsible for the expression of the various blocs. For example, it was noted that cassettes 21-24 were similarly expressed under both 30-minute oxidative stress and thermal stress whilst the expression of cassettes 1-15 differed markedly under these same stressors (Figure 7).

### Localising a promoter within the *Vibrio sp*. DAT722 cassette array

**Figure 8:**
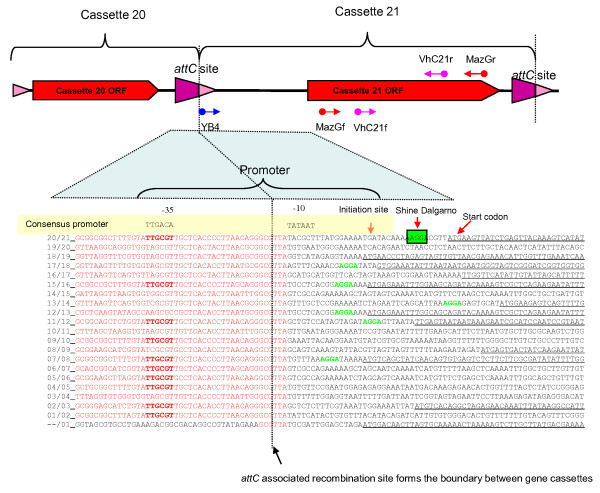
**Localising the promoter at the junction cassettes 20 and 21 in the *Vibrio sp*. DAT722 array**. The diagrammatic representation of this area of the array indicates cassette boundaries and PCR primer locations. The putative promoter is highlighted in yellow above the sequence alignment comparing the cassette junctions of the first 21 cassettes in the array. The cassette number within the gene cassette array is shown in the left-hand column. Homology with the -35 area of the cassette 20/21 promoter is shown in bold. Shine-Dalgarno sequences are shown, where they occur, in green. ORFs contained within each gene cassette are shown underlined.

It was expected that a possible location for an intra-array promoter would be indicated by cassettes that were not detectably expressed, as adjacent expressed cassettes necessarily required the presence of a promoter. Cassette 19 was not detectably expressed under any stressor while adjacent cassettes 18 and 21 were both strongly expressed, though under differing stressors. In order to identify the promoter in this region of the array, the 5' end of the cDNA transcript was localised through the PCR of nested primers bracketing the target area. An initial examination of the area between cassettes 16 and 21 (Figure 4) narrowed the target area to the region between cassettes 20 and 21. The location of the promoter was further localised as shown in Figure 8. These PCRs showed that the majority of cDNA transcripts containing cassette 21, did not include cassettes 19 and further that most cassette 21 transcripts commenced between the cassette 20 3' *attC *site and the cassette 21 ORF. The region of sequence adjacent to the cassette 21 Shine-Dalgarno sequence was examined for a possible promoter sequences in an appropriate position, with an example of a sigma 70-type promoter being found bridging the cassette 20-21 *attC *site (Figure 8). The activity of this putative cassette 20-21 promoter was tested by measuring relative amounts of transcript on either side of the promoter using QPCR. This work showed an approximately 40-fold increase in transcript on the cassette 21 side of the promoter indicating that this promoter was indeed functional. It was also noted in the QPCR work that low levels of YB4-MazGr transcript were present, indicating the presence of an additional promoter in the region of cassette 19, responsible a portion of the expression of cassettes 20-21. Consequently, the cassette 21 expression seen in the *attC *PCR was due to the presence of a transcript containing cassettes 20 and 21 and the additional expression of cassette 21 due to the cassette 21 promoter was not measured in this assay.

### Comparison of *attC *sites for promoter sequences

The cassette 21 promoter appeared to bridge the *attC *junction between cassettes 20 and 21 (Figure 8). The putative -35 site was contained within the cassette 20 side of the *attC *site while the -10 site was immediately adjacent to the cassette 21 section of *attC*. Sequence examination of other *attC *junctions within the DAT722 array showed that the position and sequence of the -35 site was present in a number of other *attC *sites within the DAT722 array. However, the corresponding -10 site within cassette 21 (Figure 8) was not present in any of the other cassette in the DAT722 array. This indicated firstly that this potential promoter could remain functional if cassette 21 were mobilised to a location with an *attC *site containing the appropriately located -35 sequence. Secondly, the -10 site, being unique to cassette 21, indicated that this promoter was unique within the array. These observations indicated that the remainder of the expression seen within the DAT722 array was due to other types of intra-array promoter. The observation of detectable but unexpressed gene cassettes adjacent to expressed cassettes in other areas of the *Vibrio sp. *DAT 722 array, suggested that additional intra-array promoters might also be located in the vicinities of cassettes 19, 35, 60, 96, 99, 106 and 108-109.

### Some implications of widespread gene cassette expression in large arrays

We have found, that in the *Vibrio *sp. DAT722 gene cassette array, the majority of gene cassette-associated genes were expressed, that this expression was largely conditional and that the expression was facilitated by multiple, different, intra-array promoters. These findings have a significant impact on our understanding of the utility of the integron/gene cassette system in prokaryotes:

Firstly, the widespread expression of cassette-associated genes within the 116-cassette array indicated that a wide range of the phenotypes implied by cassette array was available to *Vibrio sp*. DAT722 host. So, rather than being restricted to only those phenotypes that may be provided by cassettes proximal to the integron, this prokaryote lineage has the potential to benefit from all cassettes present, irrespective of their location within the array. Further, because the widespread expression in DAT722 was due to cassette-borne promoters that are themselves mobile genetic elements, it is likely that promoter-containing cassettes are ubiquitous in the gene cassette metagenome. Therefore, we concluded that cassette-associated genes within all large arrays may be routinely expressed and so, cassette arrays in general are able to confer phenotypes in proportion to their size. Consequently, the presence of larger cassette arrays can provide distinct selective advantages to the host organism and this may well account for the observed prevalence of large arrays in the environment [21].

Secondly, the presence of cassette-borne promoters indicates that these promoters as well as cassette-borne ORFs may be rearranged within the array by the action of the IntI integrase. Consequently, with the observation of polycistronic cDNA transcripts in this work and elsewhere [11], repeated rounds of rearrangement may result in the assembly of a number of tandem genes of related function within a gene cassette array, in association with an appropriate cassette-borne promoter. Such 'gene cassette operons' could result in the co-ordinated expression of multiple cassette-associated genes to produce complex phenotypes [22]. The existence of such hypothetical 'gene cassette operons' is supported by observations that differences amongst the cassette arrays of the vibrio pandemic strains were largely confined to contiguous multi-cassette indels rather than single cassette indels [20]. Similarly, the observation that a large proportion of environmental integrons have an inactive integrase gene may also be a reflection that the existence of advantageous gene cassette operons may necessitate the preservation of not only gene cassette complement but intra-array cassette order as well [23]. Further, where a functional integron-associated integrase gene is associated with a cassette array, it has been observed that the integrase gene may be induced by cellular stress [13]. This induction, enabling the recruitment of novel cassettes or groups of cassettes to the array further underscores the adaptive role of cassette arrays,

### Further research

We have established here a link between environmental stress and the differential expression of cassette-associated genes. It has also been established that lateral gene transfer involving gene cassettes can rapidly and randomly produce new phenotypes in prokaryote communities [24]. However, because of the random nature of the new arrangements of cassette-borne genes and promoters produced by LGT, the resulting novel phenotypes may not necessarily be 'finely-tuned' to the stressor that causes them to be produced. Similarly, evidence for markedly decreased translation of widely spaced genes on polycistronic cassette transcripts [25] may indicate that the ultimate outcome of the expression of individual cassettes shown in this work may not necessarily result in an advantageous phenotype. Consequently, it remains to be demonstrated, that the conditional expression of gene cassettes, as seen in this work, produces phenotypes that appropriately address the applied stressor.

## Conclusions

In this work, we have demonstrated that the majority of gene cassettes in large integron-associated arrays are expressed conditionally in response to environmental stressors and that this expression is facilitated by the presence of different intra-array promoters. These findings, demonstrate that large cassette arrays may produce diverse and complex phenotypes that are reactive to environmental changes, and so demonstrate an increased repertoire of the adaptive capabilities of the integron/gene cassette system.

## Authors' contributions

CM conceived the work, designed and executed the experiments and analysis, and co-wrote this publication. ML contributed to the analysis and co-wrote this publication. Both authors have read and approved the final manuscript.
